# Supplementary figures and images for: Comprehensive comparative analysis of the effects of temperature on the Notch signaling response in vivo
Source: Biol Open. 2025 Oct 15;14(10):bio062031. doi: 10.1242/bio.062031 (PMC12570151; doi:10.1242/bio.062031)

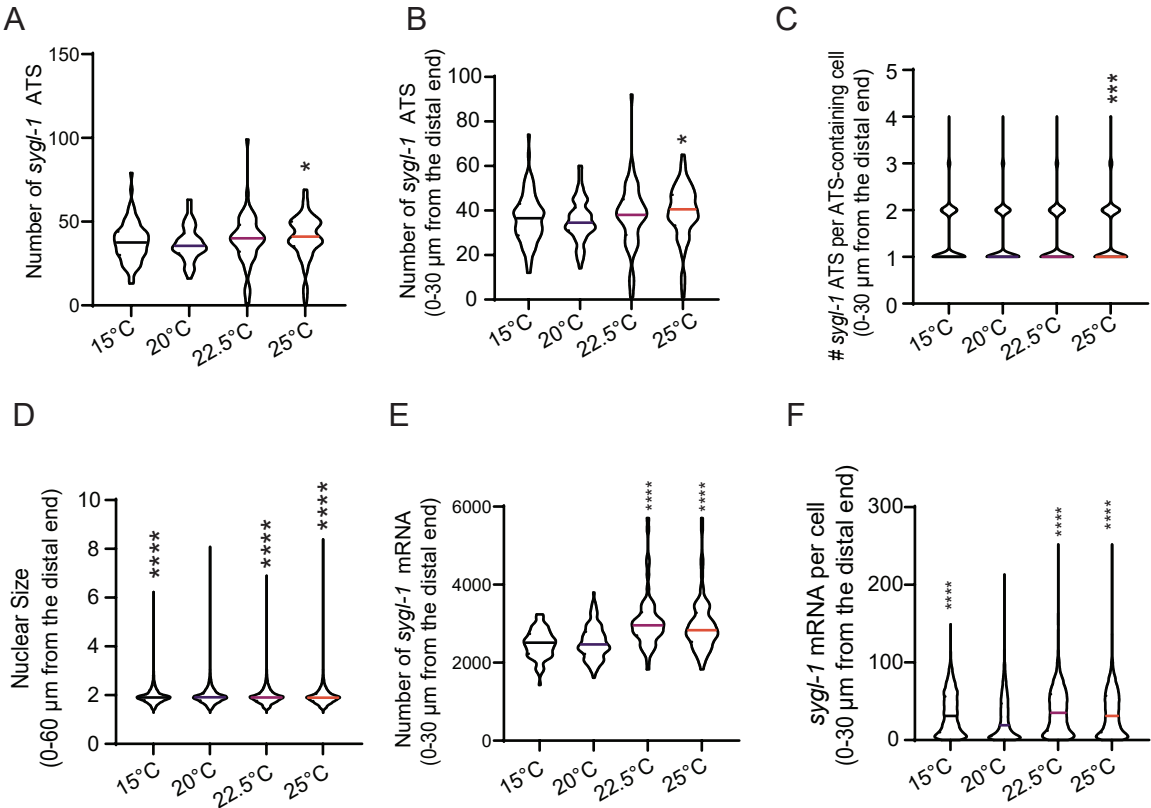

**Fig. S1.**

Supplement: Supplementary information [file biolopen-14-062031-s1.pdf]
